# A social systems analysis of implementation of El Salvador’s national HIV combination prevention: a research agenda for evaluating Global Health Initiatives

**DOI:** 10.1186/s12913-018-3667-8

**Published:** 2018-11-12

**Authors:** Julia Dickson-Gomez, Laura A. Glasman, Gloria Bodnar, Molly Murphy

**Affiliations:** 1 Center for AIDS Intervention Research, Medical College of Wisconsin, Milwaukee, WI USA; 2 Fundación Antidrogas de El Salvador, Santa Tecla, El Salvador

**Keywords:** Global Health initiatives, HIV, Combination prevention interventions, Implementation science, El Salvador, Latin America, Men who have sex with men, Commercial sex workers, Transwomen, Global Fund to fight AIDS, malaria and tuberculosis

## Abstract

**Background:**

Global Health Initiatives (GHIs) have been instrumental in the rapid acceleration of HIV prevention, treatment access, and availability of care and support services for people living with HIV (PLH) in low and middle income countries (LMIC). These efforts have increasingly used combination prevention approaches that include biomedical, behavioral, social and structural interventions to reduce HIV incidence. However, little research has evaluated their implementation. We report results of qualitative research to examine the implementation of a national HIV combination prevention strategy in El Salvador funded by the Global Fund to Fight AIDS, Tuberculosis and Malaria.

**Methods:**

We conducted in-depth interviews with principal recipients of the funding, members of the Country Coordinating Mechanism (CCM) and front line peer outreach workers and their clients. We analyzed the data using a dynamic systems framework.

**Results:**

El Salvador’s national HIV combination prevention strategy had three main goals: 1) to decrease the sexual risk behaviors of men who have sex with men (MSM), commercial sex workers (CSW) and transgender women (TW); 2) to increase HIV testing rates among members of these populations and the proportion of PLH who know their status; and 3) to improve linkage to HIV treatment and adherence to antiretroviral therapy (ART). Intervention components to achieve these goals included peer outreach, community prevention centers and specialized STI/HIV clinics, and new adherence and retention protocols for PLH.

In each intervention component, we identified several factors which reinforced or diminished intervention efforts. Factors that negatively affected all intervention activities were an increase in violence in El Salvador during implementation of the strategy, resistance to decentralization, and budget constraints. Factors that affected peer outreach and sexual risk reduction were the human resource capacity of grassroots organizations and conflicts of the national HIV strategy with other organizational missions.

**Conclusions:**

Overall, the national strategy improved access to HIV prevention and care through efforts to improve capacity building of grass roots organizations, reduced stigma, and improved coordination among organizations. However, failure to respond to environmental and organizational factors limited the intervention’s potential impact.

**Electronic supplementary material:**

The online version of this article (10.1186/s12913-018-3667-8) contains supplementary material, which is available to authorized users.

## Background

Global Health Initiatives (GHIs) such as the Global Fund to Fight AIDS, Tuberculosis and Malaria (Global Fund), the President’s Emergency Plan for AIDS Relief (PEPFAR), and the World Health Organization (WHO) among others, have been instrumental in the rapid acceleration of HIV prevention programs, HIV treatment access, and availability of care and support services for people living with HIV (PLH) in low and middle income countries (LMIC) [1, 2]. These efforts have increasingly used combination prevention approaches that include biomedical, behavioral, social and structural interventions to reduce HIV incidence. For example, most GHIs include behavioral interventions to promote condom use and HIV testing for early detection of HIV, linkage services to treat PLH and prevent onward transmission to their sexual partners, advocacy to promote the rights of sexual and gender minorities, integration of reproductive health and HIV services to prevent mother to child transmission, and integration of TB and HIV services [2, 3]. Early efforts were implemented in an emergency fashion and while many lessons have been learned through the examination of these efforts, state-of-the-art monitoring, evaluation, and research methodologies were not fully integrated or systematically performed [1, 4]. Recently, GHIs have called for more systematic evaluation using an implementation science framework to improve the development and effectiveness of their programs. Implementation science frameworks are particularly useful for understanding combination prevention interventions implemented at regional and national levels which require coordination of previously separated sectors of health systems and involvement of non-governmental and community-based health systems and social services. This paper presents results of qualitative research to examine the implementation of a national HIV combination prevention strategy in El Salvador, funded by the Global Fund.

Implementation science is commonly understood as the study of methods and strategies to promote the uptake of interventions that have proven effective into routine practice with the aim of improving health [5]. Therefore, implementation science examines what works, for whom and under what circumstances, and how interventions can be adapted and scaled up in ways that are accessible and equitable. For GHI to be scalable and sustainable, it is important to understand factors that may facilitate or impede effective collaboration among the many sectors and contexts involved in these initiatives, such as grassroots organizations that advocate for vulnerable and stigmatized populations, individual HIV clinics and the Ministry of Health [6, 7]. In this study, we analyzed El Salvdor’s national HIV combination prevention strategy using Van Olmen’s Health Systems Dynamic framework which includes the influence of the sociopolitical context, characteristics of the targeted population, leadership and governance, and resources and service delivery [6].

Implementation research on Global Fund and PEPFAR strategies has revealed that, in the early years of GHIs, disease-specific funding mechanisms have created barriers and disincentives to strengthening LMIC’s national health systems [4, 8–13] Funds were allocated with the intention of overcoming weaknesses in national health systems by establishing separate clinics that were better resourced than the national systems. In these clinics, medical staff received higher salaries, leading to a further weaking of existing health structures as medical personnel left the national systems to work in GHI clinics. Recent Global Fund efforts have attempted to align funding initiatives with national health priorities and systems [4, 14, 15], yet misalignment of activities and aims persist. A recent mixed methods study analyzing the budget allocations and expenditures in human resources for health in 27 countries that had received Global Fund monies found that there were several missed opportunities for health systems strengthening. Countries used salary top-ups, performance incentives, extra compensation and contracting of part-time workers to pay health workers using Global Fund monies. Training was in-service and disease specific. Most importantly, funding support was not coordinated with national strategic plans leading to major problems in sustainability of efforts [4].

The research conducted to date highlights that implementation of GHI programs and other interventions must be understood as occurring within a complex network of influences including the national health care system, organizational culture within particular health care centers, non-governmental organizations, and the larger political and cultural context. A dynamic systems framework can be used to understand the forces within the complex systems that affect intervention implementation, outcomes and sustainability [16–22]. Within this framework, researchers build models to understand the interactions among actors and actions, and how interventions in turn may affect and be affected by these interactions [16–22]. Models can be developed through mathematical modeling [23], and participatory or collaborative analyses with stakeholders [18, 24–26]. Another approach is to use a mixed-method, ethnographic case study design to provide detailed descriptions of the contextual factors and processes that appear to interact and make a difference in a particular real world setting and then to test the model resulting from this process in the same or different settings [16, 27].

Systems act as a functional whole composed of a set of components working together in ways that may not be apparent from the functioning of individual components [21, 23]. System level interventions require understanding systems’ components and dynamics so that we can plan and anticipate the consequences how changes in one component will affect other components with the aim of identifying leverage points that can facilitate systems level change [18, 21, 23]. For example, changes in one system component may create “feedback loops,” that reinforce an intervention’s positive effects (i.e., “reinforcing feedback loops”), or negative feedback loops that “turn off” one process when another is activated (i.e., “balancing feedback loops”), thus maintaining the status quo [23]. An example of a balancing (negative feedback loop) might be increases in sexual risk behaviors among MSM following repeated negative tests, while an example reinforcing (positive) feedback loop might include TW’s increased use of an STI clinic that has the reputation of providing trans-sensitive care.

This paper reports on results of qualitative research to examine the implementation of a national HIV combination prevention strategy in El Salvador. In March 2014, El Salvador began implementing their national strategy which included three components: 1) targeted outreach and HIV testing among populations with highest prevalence of HIV including men who have sex with men (MSM), transwomen (TW) and commercial sex workers (CSW); 2) efforts to increase linkage into HIV medical care and improve adherence; and 3) integrated social services to address the psychosocial needs of PLH. The plan differed from prior efforts in its emphasis on primary prevention, inclusion of NGOs that worked with and were led by members of the vulnerable groups targeted by the strategy, and efforts to increase coordination and decentralization. The Ministry of Health of El Salvador and Plan International, an international non-governmental organization (NGO) that engaged local NGOs to staff community outreach centers, were the principal recipients of Global Fund Monies. A country coordinating mechanism (CCM), which included the Ministry of Health, Plan International, participating NGOs and members of the affected population, monitored and governed the national strategy.

Component 1, targeted outreach, included setting up community centers that were staffed by members of the targeted populations. Outreach used a “stages of change” approach in which intervention messages were tailored to individual’s level of readiness to change their risk behavior (e.g. use condoms, take an HIV test) [28]. Component 2 included specialized clinics (VICITS) in which staff were trained to provide HIV and other STI testing in a non-stigmatizing environment. Component 3 involved the development of new protocols within HIV clinics for increasing retention in HIV care and ART adherence among PLH. Finally, the plan intended to provide for the psychosocial needs of target populations including mental health and substance use treatment, and legal advocacy for cases of human rights violations.

The main goals of the national strategy were to: 1) decrease the sexual risk behaviors of MSM, CSW and TW; 2) increase testing rates among members of these populations to increase the number of PLH who know their status; and 3) to improve linkage to HIV treatment and adherence to ART.

## Methods

To examine the implementation of the strategy and the potential direct and recursive influences on the application of intervention components we conducted in-depth interviews with program staff at different decision making and implementation levels between September 2014 and October 2015. First, we interviewed the members of the CCM(*n* = 20) including members of Plan International and the Ministry of Health, NGOs that served TW, CSW, and MSM. All members of the NGOs were also members of the populations they served. We asked participants about barriers and facilitators to initiation of the strategy, how organizations were selected to run the community outreach centers, the roles of the CCM, decision making within the strategy, and elements of the strategy that were working or not working as well as hoped. Next, we interviewed members of 4 VICITS clinics in 4 geographically separate districts (Central, East, West and Coastal) (*n* = 20). Participants included the variety of personnel who staffed the VICITS clinics including nurses, physicians, lab technicians, administrators and health promotors. Interview questions covered the nature and quality of collaborations between the VICITS clinics and community outreach centers and HIV clinics, challenges in working with TW, CSW and MSM, sensitivity training, and what they felt worked well and didn’t work well in their roles in the strategy. We also conducted interviews with personnel at HIV clinics located in four hospitals in geographically distinct regions of the country (*n* = 20). Again, participants included the variety of personnel employed in the HIV clinics including physicians, nurses, pharmacists, psychologists, social workers and health promotors. Interview questions were similar to those asked of personnel at VICITS clinics and included the nature and quality of collaborations, new strategies to increase treatment retention and ART adherence, and challenges in implementing the new strategy. Finally, we interviewed supervisors and outreach workers (*n* = 18) from 9 different community outreach centers serving in equal numbers CSW, TW and MSM. Interview topics covered included their strategies to reaching their population, challenges they encountered in their work, reactions of the target population to their outreach, and their collaborations with VICITS and HIV clinics. Interview guides are included in the Additional file 1.

### Analysis

All interviews were transcribed verbatim and coded collaboratively in Spanish by five members of the research team (the Principal Investigator of the project, a US based medical student researcher, the site project director who is a licensed clinical psychologist, and two research assistants with degrees in public health). The team collaboratively developed a coding tree that captured key themes in the interviews including barriers to implementation of the plan, new strategies, the quality and degree of coordination among organizations, parts of the national plan that they felt worked well and didn’t work well, and decision making in the processes for the plan as a whole and locally.

After interviews were coded, the team analyzed data in order to build a health systems dynamic framework. First we identified characteristics of the plan including the *key actors* in implementing the plan. These included administrators who were part of the organizations that were the primary recipients of Global Fund monies (i.e. Plan International and the Ministry of Health), the funder (Global Fund), members of the Country Coordinating Mechanism (e.g. members of NGOs that served and represented members of the target populations), directors of NGOs that were selected to form community outreach centers in different municipalities, supervisors and outreach workers of community outreach centers, VICITS clinic health personnel, HIV clinic health personnel and members of the target population. We then identified the *key settings* where the strategy took place. These included community outreach centers, community venues where MSM, TW and CSW could be found and engaged in outreach, VICITS clinics and HIV clinics. We then identified the *goals* of the national strategy and particular *intervention activities* (peer outreach, VICITS clinics, patient tracking and ART counseling) that aimed to change the socks in the desired directions. Once we identified the actors, goals and intervention components, we coded for *key sociocultural factors* that affected implementation of the strategy including stigma, the context of community violence, and the organization of health care in the country. We also identified how these produced positive and negative feedback loops to each of the intervention strategies. This model was developed and refined in an iterative process with members of the research team.

## Results

**Fig. 1:**
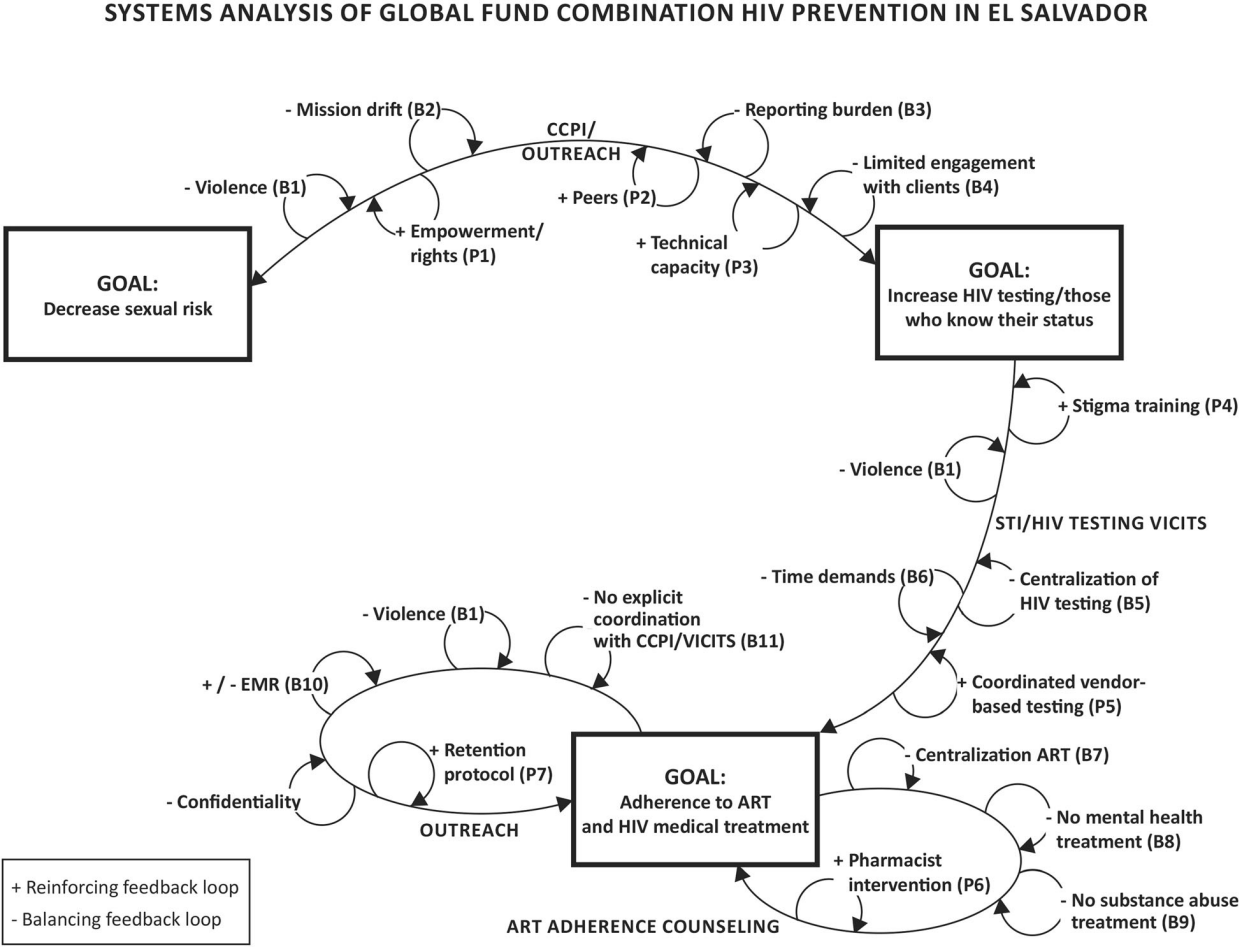
Systems analysis of Global Fund Combination Prevention Intervention in El Salvador

Figure 1 shows the goals of El Salvador’s national HIV combination prevention intervention, namely: 1) to decrease sexual risk behaviors of MSM, CSW and TW; 2) to increase HIV testing rates among members of these populations and the proportion of PLH who know their status; and 3) to improve linkage to HIV treatment and adherence to ART. Intervention components included the main actions mandated by the national strategy. Thus, the strategy involved four prevention components, two involving primary prevention and two involving secondary prevention among PLH. Component 1—to achieve the goals of increasing HIV testing and reducing sexual risk—involved the establishment of community outreach centers in seven departments (government regions) to serve the needs of particular target populations. Community outreach centers were formed by already-established non-governmental organizations which were led by members of the target populations and served the populations in question. Community outreach centers coordinated outreach activities among their staff and also had newly created physical spaces that served as drop-in centers for clients. Each community outreach center was funded to support at least two outreach workers and one supervisor. Outreach workers were tasked with visiting sites where members of the target population could be found, and providing them with a series of five prevention interventions framed by the stages of change described in the transtheoretical model. These included helping clients identify their HIV risk, distributing condoms and demonstrating their proper use, and encouraging them to take an HIV test. Goal 2 involved both the community outreach centers and the additional intervention component of establishing STI clinics (known by the Spanish acronym VICITS), which were located in primary care settings and were exclusively for the use of MSM, CW and TW. Community center outreach workers set up appointments for clients to attend VICITS clinics where they received HIV and STI testing. VICITS clinics also linked newly diagnosed PLH to care, which, together with HIV clinics, contributed to achieving the goal of increasing linkage to care an adherence to ART (Goal 3). In addition to HIV and VICITS clinics, interventions related to Goal 3 implemented in HIV clinics included a new outreach protocol to find patients who had dropped out of care and adherence education and monitoring to improve ART adherence. In all cases, decentralization and coordination of efforts were planned among community outreach centers, VICITS and HIV clinics to achieve all goals.

In each intervention component, we identified several positive and negative feedback loops, which reinforced or reduced the effects of intervention efforts. Some loops were factors that influenced all activities, for example the increase in violence in El Salvador during implementation of the strategy. Other factors, including forces against decentralization and barriers to coordination, were ubiquitous throughout the national strategy but manifested in different ways in different intervention components. When the strategy was first initiated, it was envisioned that the combination prevention intervention would include close collaboration between the community outreach centers, VICITs and HIV clinics in order to identify members of the vulnerable population and get them to accept an HIV test, STI testing and treatment, linkage to HIV care, and ART adherence. As described by a member of Plan International, community outreach centers, VICITS and HIV clinics would all work together to achieve these goals and communicate and coordinate in order to link and retain PLH in care.

Participant, Plan International: Everything about service and treatment, attention and care of people living with HIV is going to be a component that is really connected with the workings of the ART clinics. They are going to give use lists of people who aren’t adherent. They are going to give their addresses and [community outreach centers] are going to look for these people so that they go back to being adherent. This is going to be a job that is going to be very coordinated with MINSAL. So yes, they are definitely our partners, they aren’t just our counterparts I will repeat, but our natural partners in the work.

While consistent with the plans outlined in the National Strategy, this ideal was rarely achieved in practice. Barriers to coordination and decentralization were caused by a number of factors, including professional territoriality, mistrust of medical professionals and rigidity of the aims that were more or less prominent in different goals and intervention strategies described below.

### Overarching barriers to the national HIV strategy: Violence and cell phone networks

While El Salvador has had among the highest rates of homicide and gang-related violence for decades, homicide rates increased dramatically in 2014 with the change in government. Between the years of 2014 and 2016, El Salvador was dubbed the “murder capital of the world” with an average of one homicide per hour, a murder rate 22 times of that of the US, most of which was attributed to gang violence [29]. This increase in violence coincided with the implementation of the national strategy and this project. The violence was not anticipated by the Global Fund and affected every one of the three strategy goals (reducing sexual risk, increasing HIV testing and linkage to care, and increasing adherence to ART and HIV medical treatment). Outreach workers complained that the problem of violence in completing work had been underestimated and offered practical solutions, such as offering money for transportation or project owned vehicles.

Community outreach worker: We work at night…without any stipend, only $5 that we have for a taxi…to go from the community outreach centers to the business and from the business to a boarding house because we aren’t allowed to stay in the community outreach centers. The rest comes from my pocket, from my salary and so that is another of the limitations. So…this puts us at risk that they assault us, that they beat us, or even that they kill us going to places where they don’t know us, don’t even know what we’re doing there….. So this is big risk that the Plan International and the Global Fund shouldn’t put us in.

Violence affected whether PLH or those needing an HIV or STI test or follow-up care were able to make appointments as many had to travel through rival gang territories in order to reach VICITS or HIV clinics. Violence also caused many PLH or vulnerable populations to relocate, change phone numbers or otherwise hide when faced with threats from gang members. This also affected follow-up to look for PLH who may have missed appointments.

HIV Medical Provider: There are places in the country, it has to be said, that you can’t go to. You can’t access them because of the danger. In fact, our teams have been threatened many times, so while this social problem isn’t solved, this also affects adherence. There are patients who have to flee because of threats, so they abandon treatment. We try to find them, they have moved obviously, because of threats they change their phone numbers….. We can’t find them…. I am very pragmatic about these things. Are we going to have adherence with this strategy, are we going to get better, are we going to meet the goals that they want? Never, because it doesn’t depend on the service provider, or the patient, there is third factor, an external factor that the Plan, the Ministry of Health, us at a local level, the patient, or it seems, the central government cannot resolve.

Another problem that was mentioned by all providers, including community center outreach workers and health providers at VICITS and HIV clinics, was not having project-dedicated cell phones to reach clients. As is the case in many low and middle-income countries, many people who never had access to telephone service now do through cellular service. The vast majority of participants did not have access to a land line and only owned cellular phones. This presented a considerable barrier to providers, as many organizations prohibit the use of land line phones to call cellular phones due to the great costs associated with this type of call in El Salvador. In order to contact their clients or patients to find out why they have missed appointments, for example, many peer outreach workers and health care providers described using their own cell phones which constituted a significant, unreimbursed out-of-pocket expense for them.

### Activities in support of goals 1 and 2: Implementation of community outreach centers, outreach to vulnerable populations

Various positive and negative feedback loops were identified as affecting the the success of intervention activities in reaching Goals 1 and 2.

### Selection and training of organizations to be community outreach centers

#### Positive feedback loop: Capacity building and prioritization of grass-roots organizations

Organizations that were selected to run community centers were those that were made up of members of the targeted population. These non-governmental organizations were often grass-roots, run by volunteers with an activist orientation. As such, they often did not have much administrative or financial experience or capacity. Plan International, the principal recipient of the Global Fund monies, expressed that it was important to them that sub-recipients be members of the target populations and that part of their goal was to increase the human resource capacity of the organizations so that they could not only run the community centers as part of the national strategy but also solicit funds and implement projects on their own.

Member, Plan International: The criterion to select organizations was, number one, their experience in HIV work, and specifically, with the populations that we were going to target….…. Many large institutions sent us letters of intent, but many of these large institutions with lots of prestige didn’t have experience in HIV, and were eventually going to subcontract to the organizations that had the experience and many times they [the larger organizations] stayed with the administrative part and that wasn’t an objective of this grant, but rather to work directly with the institutions although they are small organizations.

Much work was done in the first year of the project to increase the capacity of smaller, grass-roots organizations to administer the large amounts of money that were provided through the Global Fund. This often included providing assistance to become legally recognized as an NGO, accounting and administrative assistance, and even help with computer skills and reporting requirements.

Member Country Coordinating Mechanism: There are administrative weaknesses in some NGOs….They don’t have administrative personnel…, an accountant or administrator. They are only activists, and when I say only activists, I don’t mean to disparage them. They know a lot about executing [interventions], but about administrative, normative and legal aspects, they don’t know anything. Another of the weaknesses or aspects that were weighed was also that some sub-recipients didn’t have adequate infrastructure…There was a specific rubric to plan how to help them even to rent an infrastructure that would permit the development of the functions if they were selected to be sub-recipients.

This technical assistance and capacity building acted as a positive feedback loop by increasing the sustainability of organizations, which in turn reinforced their abilities to provide services to their clients over time (P3, Fig. 1). Organizations that were selected felt that they had benefited from the process, as they were, for the first time, able to rent adequate space to meet the needs of the populations they were serving and pay their volunteers for their work. They also felt that being part of the national HIV combination prevention strategy gave them a respect that improved their ability to advocate for the rights of their populations with government officials, police and others (P1, Fig. 1).

Member, Community Coordinating Mechanism: Organizationally, the project has come to strengthen our ability to have employees, not just volunteers…. It has been very gratifying to be able to have a bigger space. Also, it has been a strength to have the community outreach centers working as before there didn’t exist anything like that for the population and you have a weight and a renown within the population. Before we could go, for example, to the mayor and he never received us, but now saying that there is a community outreach center that works for this and that through such and such an organization, you can go anywhere so this has opened many doors.

#### Balancing feedback loop: Mission drift

Some organizations feared that the demands of the funders would take them away from their principal activities as advocates for their populations. This is a potential balancing feedback loop labeled “mission drift” (B2, Fig. 1). As described in the introduction of this paper, the narrowing of organizational mission to the disease-specific dictates of GHIs has been observed in many parts of the world lending credibility to the NGO leader’s fears expressed below.

CCM member: Maybe they don’t see us so much in prevention, because for us, the prevention of HIV is like a transverse axis of everything that [we do]…. Maybe that was a criterion for why they didn’t select us in the end, right, but all the same, we’re not going to be sorry now, ha ha….Because if we had won the proposal… if I know anything it’s that the monkey dances for money. With money you can do a lot, but it’s another big package [HIV prevention] apart from what we have. We have many of our own missions, and we have to keep doing it. We can’t stop, because the nature of our organization from the beginning was to empower women.

The participant above was one of the founders of an organization that has advocated for sex work to be considered work like any other. This orientation has put them in disaccord with many GHIs, including PEPFAR, which periodically restricts funding for HIV prevention to groups that they feel promote prostitution rather than helping women leave sex work. These restrictions have served as barriers to community empowerment-based intervention for sex workers globally and represent the competing missions and goals of narrowly focused GHIs as opposed to the broader agendas of many NGOs [30].

#### Balancing feedback loop: Reporting burden

In spite of the increased capacity building, members of Plan International and the NGOs staffing the community outreach centers felt that the reporting demands of the Global Fund were burdensome (B3, Fig. 1). Some of the reporting may have been in order to collect accurate information for monitoring and evaluation. However, some of it appeared to be to make sure that funds were used as they should be in order to prevent misuse which came as a result of GF monies previously being misspent.

Coordinator Community Outreach Center: As supervisor I am responsible for monitoring and evaluation, making sure that the educators or agents of change have their supplies, that they give me a report of everything they are going to need for the week and give them their supplies weekly, or sometimes daily, filling out reports and making sure that the forms are filled out correctly, from the F1 that are forms of proof that the clients need to sign, they ask for the project specific ID that we work with, seeing that the F2 is done correctly, which is the form in which we record the total number of lubricants and condoms that we have distributed and the F4, which is the total number of condoms that leave storage.

This heavy reporting demand was particularly burdensome for activists of NGOs who had little computer experience and lower levels of education. In some cases, this led to the hiring of administrative professionals who were not members of the target populations. Adding administrative personnel to grass-roots organizations may have acted as a balancing feedback loop as their positions were created and paid for by global fund monies and it was not clear that this would be sustainable after Global Fund funding ended. In addition, many members of the key population who were long time volunteers of the organizations resented the presence of administrators who had no direct experience with the populations they served. Some felt that administrators lacked understanding and compassion for their populations and did not appreciate the work of outreach workers.

### Peer outreach

#### Positive feedback loop: Peer credibility and non-stigmatizing, decentralized spaces

The creation of Community Outreach Centers was seen as a positive for many outreach workers, members of the CCM and health care providers at the VICITS clinics. They saw the creation of spaces outside of the capital for vulnerable and stigmatized populations as a plus, as in the past, many NGOs that served CSW, MSM or TW were concentrated in the capital, San Salvador. This created a positive feedback loop to increase the reach to vulnerable populations. Peers educators were generally trusted as members with lived experience. Community Outreach Centers also were seen as creating safe and welcoming spaces for stigmatized populations. When vulnerable populations had positive experiences with places and peer educators sensitive to their needs, they often returned and referred others, increasing the reach of the intervention and the likelihood that they and others would receive condoms, sexual risk reduction information and HIV testing (P2, Fig. 1).

Community Center Outreach Worker: The creation of the community outreach centers, right, that have come close to the population, I am going to say that for me it’s a benefit, because now it’s not only centralized in the VICITS clinics, or physically in a health clinic, but they are out in the departments [states]. That’s an opportunity…. So having physical spaces now like the community outreach centers permits the populations to come near, without having to go to health clinics that maybe was a barrier before.

#### Balancing feedback loops: Referral to VICITS

While this decentralization of community outreach centers was seen as a strength, HIV testing was still referred to VICITS clinics rather than having outreach workers conduct rapid HIV tests with clients in the field (B5, Fig. 1). This created a balancing feedback loop as referring patients to VICITS clinics was sometimes challenging because of previous negative experiences that clients had in health care establishments. In these cases, outreach workers tried to assure clients that personnel in VICITS clinics were specially trained to treat clients with respect. Moreover, clients needed to go to the clinic, which was sometimes far away and/or impossible to get to because of gang violence as described above. In addition, the national strategy called for a very thorough physical exam, including STI testing and answering lengthy questionnaires.

CCM Member: Later there were difficulties because the system that we gather the information, a digital system, it has a questionnaire of, I don’t know, like 50 questions. It takes about an hour, the interview, so when they arrive to one of the establishments, the only doctor that is there to attend them has two or three people and another inside. This means that the person who comes has to wait two or three hours.

#### Balancing feedback loop: Limited engagement with clients

Another barrier faced by community outreach centers that may have counteracted the trust they were able to build as peers to clients were the inflexible goals of the national strategy. Outreach workers were mandated by the intervention to visit clients five times in order to give them the “complete package” of prevention services. However, once a client had received these services, outreach workers were mandated to find other members of their target populations to begin and complete the process with them. This led many community outreach center educators and their clients to feel that they were abandoning people who still faced considerable HIV risk.

Educator Community Outreach Center: There is a little bit of disagreement…that the program set goals for a certain period of time and the person should get a specific package….As an organization we don’t have a reserve of condoms. So, like the project figured 144 condoms to complete the cycle, but what happens when the girls have gone through the cycle and we already gave them their 144 condoms. There is some unhappiness, for example, in saying “Look. Why didn’t you invite me to the meeting anymore?” And then you have to explain, “Well, now we are with other new people.” “Oh, okay, but at least give me some condoms or some lubricant or something.” So, for this year I have understood that there are going to be follow-up supplies…however, I don’t know how many they are going to have for each user. I would like them to, at least leave them in a warehouse because the girls are always coming to ask for them.

The lack of necessary supplies acts as definite negative feedback loop to the goal of decreasing sexual risk.

### Activities in support of goal 2: Implementation of VICITS clinics to increase HIV testing

#### Positive feedback loop: Anti-stigma training and culturally competent care

VICITS clinics were designed to give priority treatment to populations vulnerable to becoming infected with HIV including MSM, TW and CSW. This included having dedicated lines and waiting areas for members of these target populations so that they might avoid the stigma, loss of confidentiality, and longer wait times they would confront if they were to wait in the same lines as the general patient population for an appointment. Personnel at VICITS clinics also received sensitivity training in order to treat patients in non-stigmatizing ways, such as using the preferred pronouns and names of TW.

Outreach workers and clients at community outreach centers reported that ongoing training had reduced stigma at VICITS clinics and, as a result, clients were more willing to accept an HIV test than at the beginning of the strategy (P4, Fig. 1). Outreach workers explained that many clients felt they had been mistreated at health care facilities in the past which played into their reluctance to go to the clinic for an HIV test until word of mouth assured them that the treatment they would receive was “caring and of high quality.”

Educator MSM: In the case of the HIV test there was resistance at first…That has changed and it has helped us a lot that we are working with and referring people to friendly spaces, culturally sensitive where they are going to be received and treated well. That was a lot of the reason that many people had bad experiences in getting an HIV test and because of that bad experience they didn’t ever want to go back much less try to convince others that they do it. Now it’s different. Because of the outreach we have done, training workshops sensitizing health personnel and other entities, when a client goes for services, if he goes alone or accompanied by an educator, the treatment is the same and it’s good.

This resulted in a positive feedback loop, where good experiences at VICITS clinics were reported to others, increasing others willingness to accept an HIV test.

Community center outreach workers, as members of the populations they served, were responsible for training VICITS clinic personnel in anti-stigmatizing and non-discriminatory care. Because they were also in close and continuous contact with members of the target population and often accompanied clients to their VICITS clinic appointments, they also were able to closely monitor the quality of treatment their clients received. When they heard complaints or observed mistreatment of their clients, they made official complaints to the VICITS clinics and the CCM, and held further trainings to improve care, creating a positive feedback loop.

Community Center Outreach Worker: It isn’t easy when we are talking about sensitizing health personnel. I can be in a sensitivity training for a whole week. That’s not going to guarantee that I am sensitized about the topic they are talking to me about…. So, what we try to do is to monitor these clinics that have received training to see if the work that they are doing is worth anything and if not, we will train them ourselves and we will file complaints against them ourselves, because it is our work and our commitment with the populations.

Interventions to reduce stigma were only partially effective, however. Some health care workers had deeply held religious beliefs that made them view homosexuality and sex work as sins. Adding to the problem was the policy of rotating nurses throughout the Ministry of Health clinics every 3 months, so that new nurses were constantly being introduced without necessarily having received sensitivity training. Many VICITS providers felt that sensitivity trainings needed to be ongoing for these reasons.

#### Positive and negative feedback loops: Coordination and (De)centralization

The ways in which community outreach centers and VICITS clinics moved toward decentralization of HIV testing through coordinated venue-based HIV testing for target populations acted as a positive feedback loop in some locations. In others, HIV testing in community locations coordinated by VICITS and community outreach centers was not pursued. In these cases, gang violence often made accessing VICITS clinics impossible and referral networks had broken down or wait times in VICITS clinics made them an undesirable resource for target populations. In these locations, the continued centralization of HIV testing in VICITS clinics acted as a balancing loop.

While just two community outreach centers and one VICITS clinics reported coordinating community testing events in which they tested CSW using rapid HIV tests in the venues in which they worked, these efforts were very successful and may have helped overcome mistrust of medical providers more quickly than other VICITS clinics.

VICITS personnel: We also go out at night to some of the workers who are on the street to give them [HIV] tests. They [community center outreach workers] send them to us, follow-up with them…They receive their medical treatment much more rapidly, the attention is more personalized and they give them all kinds of tests, and if something comes out positive in one of their exams they follow-up, by telephone or by going out to the street to find them.

These coordinated visits with community outreach centers and VICITS also allowed VICITS medical care to look for patients who had not completed their STI treatments or to give them results of diagnostic tests. Furthermore, decentralization of HIV testing by allowing community center outreach workers to conduct rapid tests in venues where vulnerable populations were found might have eliminated some of the barriers to going to the VICITS clinics such as having to pass through rival gang territories. This remained an unrealized potential to increase HIV testing among those still unwilling or unable to go to VICITS clinics because of their locations in rival gang territories in most areas of the country (B1, Fig. 1).

Peer referrals from community center outreach workers to VICITS helped to establish trust and eliminated some of the long wait times that characterized some of the first months of VICITS implementation.

Interviewer: Is there coordination between the community outreach center and the VICITS clinics?

Community Center Outreach Worker: Yes. It works really well because we work based on appointment times that they give us…So what we do is I go to a bar and a girl tells me, “Look, I really need an HIV test, tomorrow I can go to the VICITS clinic.” I make an appointment and then I tell the girl….When they go with an appointment, it’s certain that they will be seen.

In other cases, communication between some VICITS clinics and community outreach centers broke down completely and members of the key populations were not going to VICITS clinics, mainly because community outreach center educators no longer referred them.

VICITS Personnel: What I would see that we are doing badly as a strategy is the fact that the organizations know about the [VICITS] clinic, they know where we are, know how we work and know what populations we serve and the services we offer. However, the organizations are working hard, I personally think, working to meet their objectives and goals and they are leaving to one side what is the principle of the strategy which is to close the cycle, give them attention in all areas….. For example, not to mention names, but [an NGO] is seeing a huge number of sex workers but how many have we received to date? Very few. If an organization tells me we see 200 or 300 people monthly, I congratulate them but of those 200 people that they have seen, we have maybe seen on three, one, four, five.

While the medical provider above places the blame on the community outreach workers for not sending them their population, the quotes above demonstrate that key population members’ decisions to undergo an HIV test or undergo a physical exam depended not only on the community outreach center educators but also on the quality of attention they received at the VICITS clinic, including feeling if they were discriminated against or not, and the time consultations took. The time that consultations at VICITS clinics took was particularly problematic for commercial sex workers, who often needed to ask permission from the owners of the businesses in which they worked (B6, Fig. 1). Time spent in the clinic was also time that they were not able to earn money through sex work.

Other community outreach centers ended up using private clinics and laboratories because they were unable to solve the problem of long waiting times at VICITS. The long waiting times made their clients reluctant to use the VICITS clinics, and prevented the community outreach centers from reaching the target number of HIV tests set for them by the funder.

#### Balancing feedback loops: Health system limitations

Other limitations that decreased the effectiveness of VICITS clinics were the lack of adequate space in many of the health facilities in which VICITS clinics were housed, which limited privacy and the ability of health care workers to provide HIV prevention education. Other resource limitations at the VICITS included stock outs of medications to treat STIs and no contraceptives for sex workers. Some laboratories complained of not always having reagents to perform STI tests.

### Activities in support of goal 3: Implementation of improved engagement and retention in HIV care, and ART adherence

#### Positive feedback loop: HIV clinic rapid response to patient no-shows

In the new national strategic plan, HIV clinic personnel are much more proactive in efforts to retain patients in care (P7, Fig. 1). Previously, patients were considered to have abandoned treatment if they did not show up for any medical appointment for three months, but in accordance with the national strategy, HIV clinics now attempt to contact patients immediately if they did not show up for one scheduled appointment.

HIV Clinic, Pharmacist: ….Patients who don’t come, those files go immediately to social work. Social work is in charge of calling the health clinics to send someone to look for that person in the place they live. As soon as the patient comes in, even if it is the second day or a month later that they found him, or six months or a year after, that patient immediately goes to the psychologist to see why he stopped treatment. He goes to the social worker and the health promotor, and to work on adherence he sees me, because I need to know why he abandoned treatment. Sometimes it’s because he has a new partner and so that his partner wouldn’t find out, he didn’t come to his appointment. Many times they get depressed and don’t want to come maybe because their partner died, or their mom died, or he lost his job, or any reason. So I need to know what happened in that moment to cause the person to abandon treatment and when we find them again, we give them appointments for six consecutive months and we are working on the adherence every month.

This early search and intervention with patients who missed appointments improved the goal of adherence to ART and HIV treatment.

HIV personnel (social workers and nurses) used a number of strategies to locate patients who missed appointments, including trying to contact them by telephone or, because many times cell phones were no longer in service, sending health promotors out to find them. Many personnel at HIV clinics mentioned that one of the challenges to conducting outreach on their own is that patients often give false addresses to protect their confidentiality. In addition, because the catchment areas of HIV clinics are much larger than those of primary care or even VICITS clinics, HIV health promotors are not always familiar with the locations where patients live, increasing their risk of violence as discussed above.

#### Balancing feedback loop: Lack of coordination with VICITS clinics and community outreach centers

Part of the problem HIV clinics had in locating clients stemmed from their lack of coordination with, and even knowledge of, the work of the community outreach centers and VICITS clinics (B11, Fig. 1). Many HIV clinic personnel had heard of some of the NGOs that formed community outreach centers but were unfamiliar with community outreach centers work. As a result, coordination from community outreach centers to VICITS clinics and VICITS to HIV clinics occurred as planned with community outreach centers bringing patients to VICITS clinics, with some exceptions where the relationship between community outreach centers and VICITS clinics appeared to have been broken. The relationship between VICITS clinics and HIV clinics also appeared to work well with VICITS as the referring organization. VICITS clinic personnel reported accompanying patients who tested positive for HIV to their first appointment at the HIV clinic, thus facilitating linkage to care. They talked about this being a way of ensuring that linkage had, in fact, occurred.

VICITS Clinic: We don’t let a patient go alone to the [HIV] clinic. They always go with a health professional to make sure that they went to the ART clinic.

In contrast, HIV clinics were much less likely to use VICITS clinics or community outreach centers to locate participants who had missed appointments with a few exceptions. This was in spite of the fact that community center outreach workers and some VICITS workers already knew where patients could be found, were familiar working in the areas, and had established a great deal of trust and despite the fact that this coordination had been envisioned as part of the strategy. Most HIV clinics contacted local primary health care clinics to help find patients lost to care rather than community outreach centers or VICITS clinics. Although these clinics were decentralized and personnel were more familiar than the HIV clinic personnel with the locations where patients lived, HIV clinic personnel reported that some clinics were willing to help locate patients while others were not, citing fears of being sued for violating patients’ confidentiality.

Participant HIV clinic**:** The coordination that we have with the primary care center is to find and look for patients who miss refilling their prescriptions, their medical appointments and their labs. It’s very difficult because not all the health clinics collaborate with us. There are health clinics that we work with very, very well. There are some health clinics that are hard, because I think that still they need to educate them more…that the information they have is maybe not the most accurate. Some nurses tell me that we can’t send them to look for patients “Because I am not going to get involved in a problem of a law suit” so they block me.

It appears that the strategy had not conducted outreach with primary care clinics or that there were no protocols in place, such as Releases of Information (ROI), that would ensure patients’ consent (or lack of consent) to the use of other medical personnel to locate them. Such protocols could have protected the confidentiality of PLH who may have feared status disclosure in outreach efforts as well. It may have been easier to establish joint ROIs with VICITS and community outreach centers who were already familiar with the national HIV strategy, so HIV clinics’ failure to do this is surprising. The failure of HIV clinics to reach out to community outreach centers and VICITS clinics to locate patients is an untapped resource that acted as a balancing feedback loop to the goal of improving HIV care and ART adherence.

#### Positive and negative feedback loops: Electronic medical records

As part of the national strategy, the Ministry of Health developed a new electronic health information system that recorded PLHs medical appointments, missed appointments, and lab results. This database could be shared across all HIV clinics in the country, and also included information about incarceration. Some HIV clinics reported using this health information system and that it greatly facilitated their efforts to locate patients who have dropped out of care. Other HIV clinics did not appear to be using the health information system to its full potential—or at all—and still relied on paper patient records which were stored outside of the HIV clinic. Lack of knowledge and training in all the new systems, including the new electronic medical records, acted as a barrier to their use at all HIV clinics and served as a negative feedback loop in active outreach to get patients who had been lost into HIV treatment (B10, Fig. 1).

#### Balancing feedback loop: Centralization of ART

One of the barriers to improving ART adherence was that medications were still centralized within the HIV clinics (B7, Fig. 1). Each HIV clinic had its own pharmacy and pharmacist separate from the rest of the hospital which allowed patients to receive their medications in relative privacy and allowed the pharmacists to intervene with them to improve their adherence. One pharmacist talked about plans to decentralize medications to primary care clinics for patients who had been on ART for a number of years and had achieved 100% adherence levels. Other pharmacists talked about health promotors delivering medications to patients who were very ill or who lived some distance from the HIV clinic. However, many pharmacists were reluctant to decentralize medications, expressing concerns regarding patient confidentiality and poor medication adherence.

Participant HIV clinic: No, no decentralization in the sense that you are going to go to a health clinic or some other place, right. Understand that the basic regimen of antiretrovirals is for hospital management where there are specialists. To give them out at some local health clinic means that we would lose the anchor to measure adherence.

These concerns and reluctance acted as negative feedback loops, slowing changes toward decentralization. In addition, the practice of using primary health care facilities to help locate patients who have missed appointments contradicts the confidentiality concerns around decentralization of HIV medications.

Pharmacist HIV clinic: The thing is, it’s here, the medication is here in the hospital. We have it. We can’t decentralize it. The Ministry hasn’t yet told us anything about that because many times if you send it to the health clinics there is a lot of discrimination….They need to be educated so that they won’t go and say Don So-and-so is….[HIV positive] Sometimes we don’t send promotors from the health clinics [to find patients] because they gave us problems. We had a problem in Tacuba and after we said that they need talks about not discriminating because the promotor told [that the patient had HIV], so now better not to, better send our promotors that know that there is confidentiality about that.

#### Positive feedback loop: Pharmacist interventions to improve adherence

In spite of its drawbacks, centralization of medications did allow for pharmacists who were experts in treating patients with HIV to work with patients to monitor adherence and intervene to improve adherence when necessary. This was a potential positive feedback loop as detection of nonadherence and intervention could help improve overall adherence (P6, Fig. 1).

#### Balancing feedback loop: Lack of mental health and substance abuse treatment

A final barrier to improving adherence to ART and HIV medical care is the lack of coordinated services for those suffering from mental health or substance abuse problems (B8 and B9, Fig. 1). While each HIV clinic had psychologists and social workers, these were trained only to deal with crisis situations, such as loss of housing, and help improve ART adherence. Most reported that they had not received specialized training in substance abuse, and did not feel capable of treating patients with serious mental illness [31]. Many clinics had no psychiatric or substance abuse treatment systems on site. In these cases, they often referred patients to the Psychiatric hospital in San Salvador. However, most HIV clinic psychologists and social workers realized that these referrals were not effective because patients and their families found going to the psychiatric clinic, which was located in another hospital, stigmatizing.

In only one HIV clinic, there were on-site mental health and substance use services, which helped overcome the problems with referring patients to hospitals or sites far away. However, even in this case, personnel reported that coordination between those treating mental health and substance use and the HIV providers was less than ideal.

Participant HIV Clinic: Here we have a doctor in the hospital who was trained last year, I think, he got a certification in addictions. So, we are referring patients to him who have some sort of addiction. The problem is that it is just him for the whole state, so all the local health clinics also refer patients to the mental health clinic for him to see, in addition to the one we get here. Another problem is that I think that a person who sees people with addictions should also have some training in HIV in antiretroviral therapy because there are drugs that can cause interactions with the antiretrovirals. In addition he should know about the medication adherence because we can’t talk about addictions without knowing about adherence. So that’s what complicates things, because he is treating addictions in isolation and the [HIV] clinic is trying to given integrated medical attention.

## Discussion

El Salvador’s national HIV prevention strategy made progress toward many of its goals and also avoided some of the pitfalls of previous GHI strategies. One of the major goals of the national strategy was to strengthen the capacity of grass-roots organizations working with and represented by members of vulnerable populations. Grass-roots organizations were offered assistance in becoming legal entities and in fiscal and administrative management of budgets that were considerably larger than what they had previously been awarded. NGOs that administered community outreach centers appreciated the increased capacity and the credibility that being part of the national strategy afforded them. They reported being able to pay staff and expand physical spaces to work directly with clients, many for the first time. They were also able to meet with government officials and health care workers to advocate for their clients’ rights and felt, again many for the first time, that their voices were heard. Likewise, efforts to increase HIV testing among vulnerable populations, linkage and maintenance to HIV medical treatment and ART adherence were implemented within the existing Ministry of Health clinics. VICITS clinics were located within primary health care clinics but were for the exclusive use of the vulnerable populations. Likewise, new efforts to maintain patients in HIV care and improve ART adherence were implemented within already existing HIV clinics. Working with and strengthening existing health and advocacy organizations avoided the creation of parallel services within El Salvador and improved chances of sustainability.

Efforts to increase NGOs capacity to manage HIV prevention projects of the magnitude in the national strategy were only partially effective, however. Many community outreach center personnel complained of high reporting burdens. Many NGO founders and staff were not well educated and had little experience in computing, adding to the reporting demands. In some cases, this led to hiring people with technical expertise who did not necessarily have experience with the populations served causing some resentment among more long-term staff. Other NGO staff suggested that the narrow focus of the Global Fund on sexual risk reduction would divert resources from other organization missions such as recognition of sex work as legitimate employment and economic empowerment of CSWs.

Our systems analysis of implementation revealed many positive feedback loops. The establishment of community outreach centers that were run by existing grass-roots organizations with long histories of working with and advocating for the target population and peer outreach helped establish trust among the communities of MSM, TW and CSW, which in turn encouraged other members of key populations to refer peers to receive HIV prevention services. This was somewhat undermined by a rigid protocol that proscribed continuing to work with “old clients” that had received the full prevention package. Particularly problematic was the failure to continue to provide condoms which severely undermined the goal of reducing sexual risk.

The trust established by community center outreach workers was capitalized on to refer clients to receive HIV and STI testing at VICITs clinics. Community center outreach workers acted as advocates and watchdogs at VICITS clinics, improving quality of care through continuous sensitivity training and monitoring. This, in turn, increased demand for HIV services (positive feedback loop). VICITs clinics that earned the trust of patients were able to accompany PLH to link them directly to HIV care. New protocols in place to search for PLH who missed appointments helped keep PLH in care.

Along with positive feedback loops that reinforced intervention strategies to achieve national prevention goals, there was a number of balancing feedback loops that served to impede progress. Decentralization was not achieved in many cases, even when originally intended. HIV testing in venues where vulnerable populations could be found only occurred in one location in which the community outreach centers and VICITs clinic personnel worked together to provide venue based testing. Otherwise, community center outreach workers made referrals to VICITs clinics. Efforts to increase attendance to VICITS clinics were only partially effective because VICITs clinics provided physicals that took too much time for CSW, MSM and TW. Because of the inflexibility in the length of the physical examinations, some community outreach centers began using private laboratories for HIV testing among their clients.

Antiretroviral therapy was even more centralized than HIV clinics as it was only provided in the 20 HIV clinics in the country. HIV clinic health personnel expressed much resistance to decentralization including fears about patient confidentiality and the inability to monitor adherence. This created a negative feedback loop on adherence as some patients needed to travel sometimes long distances. Also, there were few resources for PLH with substance abuse or mental health problems that may have affected adherence.

The protocol to increase linkage and retention to HIV care was undermined by a lack of informed consent from PLH for outreach efforts in order to protect their confidentiality, and the under-utilization of community outreach centers or VICITs clinics to help locate patients lost to care. In no cases were patients asked to sign Release of Information forms to specify who HIV providers could contact, and who they could not, allowing PLH more control over potential disclosure. The fact that many PLH provided false addresses and phone number indicates that concerns about confidentiality and stigma were still quite present for PLH. Consultation with PLH on ways to increase retention and provide outreach in ways that would not disclose their status could have helped overcome some of these barriers.

Coordination of intervention efforts appeared to flow in one direction, from community outreach centers to VICITS to HIV clinics, but not in the reverse. This is puzzling since community center outreach workers were most familiar with the populations most affected by HIV and the locations where PLH may be found. Many HIV clinic personnel appeared to be ill-informed about community outreach centers or VICITs clinics and how they were contributing to the national protocol. They thus relied on local family health clinics to contact patients, who did not always comply with requests and were not trained to address the needs of populations vulnerable to HIV infection.

The most pervasive negative factor influencing the implementation of all components of the national strategy was the context of violence in El Salvador. Outreach workers found it too dangerous to travel to some locations to engage members of their target populations in outreach efforts and often reported spending money out of pocket to lessen the risks of their work, for example, by paying for taxis rather than relying on public transportation. Some members of the target populations were not able to travel to VICITS clinics to take HIV tests because it required them to travel through rival gang territories. This was also the case for PLH who missed appointments at the HIV clinics. Violence also affected HIV and VICITS clinics’ ability to follow-up with “no shows” because of the danger associated with looking for patients in neighborhoods where the health workers were not known and also because of the mobility of the population who often moved due to threats from gang members. The level of violence and the impact it would have on all components of the national strategy was not anticipated by the Global Fund and no adjustments were made to adapt to this changing context, although several practical solutions (providing cell phones, providing project transportation) were suggested by front line workers.

## Conclusions

Results from this study show the importance of implementation science and the utility of systems analysis in evaluating combination prevention interventions that involve multiple organizations from different sectors. This kind of analysis is a useful tool to identify and help solve implementation challenges such as those identified here. Particularly if conducted during implementation and in collaboration with funders and service providers, a systems analysis may have resulted in important adjustments being made to address some of the challenges outlined here.

## Additional file

Additional file 1: Key informant interview guides. These are the guides used for CCM members, community outreach center staff, and STI and HIV center staff. (DOCX 22 kb)

## Abbreviations

AIDS: Acquired immune deficiency syndrome
ART: Anti-retroviral therapy
CCM: Country Coordinating Mechanism
CSW: Commercial sex workers
GF: Global fund to fight AIDS, tuberculosis and malaria
GHI: Global Health Initiatives
HIV: Human immunodeficiency virus
LMIC: Low and middle income countries
MINSAL: Ministry of health, El Salvador
MSM: Men who have sex with men
NGO: Non-governmental organization
PEPFAR: President’s emergency plan for AIDS relief (United States)
PLH: People living with HIV
PMCT: Prevention of mother to child transmission
ROI: Release of Information
STI: Sexually transmitted infection
TB: Tuberculosis
TW: Transgender women
VICITS: Clinica de vigilencia centinela de las infecciones de transmissión sexual (STI Clinics)
WHO: World Health Organization
