# Supplementary material for: A social systems analysis of implementation of El Salvador’s national HIV combination prevention: a research agenda for evaluating Global Health Initiatives
Source: BMC Health Serv Res. 2018 Nov 12;18:848. doi: 10.1186/s12913-018-3667-8 (PMC6233270; doi:10.1186/s12913-018-3667-8)
Supplement: Supplementary file 1 — Key informant interview guides. These are the guides used for CCM members, community outreach center staff, and STI and HIV center staff. (DOCX 22 kb) [file 12913_2018_3667_MOESM1_ESM.docx]

**Global Fund/International Plan Combination Prevention Intervention Strategies**

**In-Depth Interview Guides ENGLISH**

Interviews with key informants and members of the Country Coordinating Mechanism (CCM), Executive Committee, HIV medical providers, providers of supplemental services, project coordinators and outreach workers; as well as interviews with vulnerable populations including MSM, transgender women and sex workers.

**Interview Guide for Vulnerable Populations**

Interview Guide (MSM, transgender women, sex workers) **that have had contact**

1. Can you describe your typical day? (From when you get up, to what you do for a living, who you meet and communicate with, places you frequent, drug consumption)?
2. How big of a problem is HIV in your community? (Referring to MSM, transgender women and sex worker communities)
3. Do you know anyone with HIV?
4. What activities put people at risk for contracting HIV?
5. Before visiting the community centers, were you aware of other places that support HIV populations? Have you been to any of them? Which ones? What experiences did you have in these places? What care, attention, services, or information did you receive?
6. Have you had contact with a community center health promoter? Did they give you any information? What type of information did you receive? Where did the encounter take place? What happened in this encounter? Was it individual contact with the promoter? What did he/she offer you? (condoms, materials, HIV test, etc.) Were there other people besides you? Who were they? How did you know them and/or what was you relationship with them?
7. How did you feel in this moment of contact? Good things/bad things? Was the information you received useful? Did you think anything was missing? Any suggestions? Do you think the form in which you were approached was appropriate? Would you change anything about it?
8. How many times did you meet with the promoter? How has your relationship with this person? Did you know them before?
9. What do you think about the work the promoters do? Why do you think they do it? Do you know who founded and organized this type of intervention? Why do you think they did it?
10. Has your life changed after this contact? In what way? Your way of thinking, your behaviors, or anything else?
11. Have you taken any of the HIV tests offered by the promoter? Are you willing to share the test result?
12. Have you received any treatment orientation? What kind? Have you begun treatment? If yes, why? If no, why? Have you continued/follow-up? What type? Have you been contacted to continue treatment? Have you accepted the tracking? Why or why not?
13. Do you know anyone who is in need, but has not been approached by the promoters? Do you know anyone who is unwilling to talk to the promoters? For what reasons? What do you think of them?
14. Are there any other needs that are not covered by or offered by the promoters? What are they? (housing needs, food, drug consumption, mental health, violence, etc?)
15. Do you have references for any other resources or organizations that cover these needs? Have you had contact with them? Why or why not?

Interview Guide (MSM, transgender women, sex workers) **with NO contact**

1. Can you describe your typical day? (From when you get up, to what you do for a living, who you meet and communicate with, places you frequent, drug consumption)?
2. How big of a problem is HIV in your community? (Referring to MSM, transgender women and sex workers)
3. Do you know anyone with HIV?
4. What activities put people at risk for contracting HIV?
5. Have you received any information, had any contact with, and/or aware of places that support HIV populations? Have you been to any of them? Which ones? What experiences did you have in these places? (what attention, services, or information did you receive?
6. Are you familiar with combination prevention interventions? What have you heard about these programs?
7. Have you had contact with a community center promoter? (IF “YES”, THEN CONTINUE) Have they given you any information? What type of information did you receive? Where did the encounter take place? What happened in this encounter? Was it individual contact with the promoter? What did he/she offer you? (condoms, materials, HIV test, etc.) Were there other people besides you? Who were they? How did you know them/ what was you relationship with them?
8. How did you feel in this moment of contact? Good things/bad things? Was the information you received useful? Did you think anything was missing? Any suggestions? Do you think the form in which you were approached was correct? Would you change anything about it?
9. How many times did you meet with the promoter? How has your relationship with this person been? Did you know them before?
10. What do you think about the work the promoters do? Why do you think they do it? Do you know who founded and organized this type of intervention? Why do you think they did it?
11. Has your life changed after this contact? In what way? In your way of thinking, your behavior, or anything else?
12. Have you taken any of the HIV tests offered by the promoter? Are you willing to share the test result?
13. Have you received any treatment orientation? What kind? Have you begun treatment? If yes, why? If no, why? Have you continued/follow-up? What type? Have you been contacted to continue treatment? Have you accepted the tracking? Why or why not?
14. Do you know anyone who is in need, but has not been approached by the promoters? Do you know anyone who is unwilling to talk to the promoters? For what reasons? What do you think of them?
15. Are there any other needs that are not covered by or offered by the promoters? What are they? (housing needs, food, drug consumption, mental health, violence, etc?)
16. Do you have references for any other resources or organizations that cover these needs? Have you had contact with them? Why or why not?
17. (In the case that they have **NOT** been to a center) Why have you not gone? Have you every thought about going? Do you know anyone who has gone? How many people? What have they told you about it? What would your friends think if you went?

**Interview Guide for Medical Service Providers**

1. Are you familiar with the new combination prevention intervention strategies?
2. Have you received any training related these strategies? If so, was the training centered on specific populations?
3. What’s your opinion of PLH who consume drugs? Or your opinion of MSM, transgender women, and sex workers?
4. What new services are being offered to PLH that are drug users or have mental health illnesses? Are you coordinating with any other entities that offer services or support drug users or those with mental health illnesses?
5. Have you changed any retention practices for PLH to improve adherence in relation to the implementation of the national strategy?
6. Have you expanded any programs or activities to reach this population? (support groups, decentralized healthcare?)
7. Are you collaborating with other organizations? For how long? How is it going? What are the goals or objectives?
8. What communication and linkage system do you use with those that have been diagnosed in the community centers? What works or doesn’t work with this system?
9. How do you approach those who have left/abandoned treatment? What retention strategies do you use to keep them in treatment? What would happen if someone referred to the center did not show up for treatment?
10. Do you coordinate with the community centers? What type of, or what is the nature of coordination? Is there coordination for cases of treatment abandonment? What type?
11. How have you seen treatment adherence function with this new strategy? Have there been any changes? What types? What is the difference? Are the better strategies? More effective ones? Which ones, what are they?
12. What populations (MSM, trans, sex workers) are most challenged with linkage to care, ART maintenance and adherence? What are the reasons?
13. What needs are not covered?
14. Do you have a relationship with the CCM? What type? How do you coordinate?

**Interview Guide for CCM and International Plan Personnel**

1. How did you become a member of the CCM? What role do you have within the CCM? What experience did you have before?
2. What do you know about the development of the combined prevention intervention strategy? How did they arrive at creating this strategy? Was it an easy or hard process to agree on how to establish the strategy? How did they obtain information and opinions about/from MSM, trans, and sex worker populations?
3. What elements have helped facilitate, or conversely, have challenged the process of developing the plan?
4. What criteria were used to select the organizations to work in implementation and development of combined prevention plan? What aspects are considered necessary to resolve any weaknesses of these organizations?
5. Could you tell me what the new strategy consists of? What are the components? How does it differ from previous HIV prevention and treatment interventions? Is there is difference in the target population? What’s the difference?
6. Are the needs of the PLH population that are not yet covered? Or needs of other vulnerable populations, such as MSM, trans, sex workers, that are not covered?
7. What role does the CCM play within the new strategy? How are the CCM involved organizations coordinated? Is there any type of process or results evaluation? What type?
8. Is there any communication between the CCM and the affected populations?
9. How are differences in opinions or priorities resolved with each organization?
10. Have any problems arisen in implementation of the Plan? What type of problems? Do you think they have been resolved? How were they resolved? How is the plan functioning? What has been learned? Important changes made? Anything you were not expecting to encounter? What were they?
11. Is there coordination between the CCM and MINSAL? With what purpose? And with the community centers? With what purpose?
12. Up until now, what has been memorable? A specific achievement?
13. Have changes from the initial plans occurred? What are they? Reasons for them?

**Interview Guide for Community Center Health Promoters and Supervisors**

1. Are you familiar with the new combination prevention intervention strategies?
2. How were you recruited? What prior experience do you have? Were you already working with the targeted population of the International Plan? Have you worked with people living with HIV?
3. What motivated you to work on this project?
4. Have you received training in this area? What did the training consist of?
5. How has the strategy worked/ been employed in the field? Challenges? Have you had to change or implement new or different strategies?
6. Why did you choose, or how did you select specific sites for the work of the International Plan?
7. How has your experience been in this work? Were you already familiar with the location where you work? How do you feel about working in this field, with this issue area?
8. Were you working previously with this organization? Or is this a new job, new work to you?
9. What have you witnessed in the population in this location concerning the Plan?
10. What are the defining characteristics of the population in general, in this location where you are working with the Plan?
11. Has it been easy or challenging to get to know MSM, trans and sex worker populations? For what reasons?
12. What attributes or situations specific to the location have affected development of the program?
13. Have you observed examples of stigmatization in relation to these populations (MSM, trans, sex workers)? Have improvements on stigmatization been achieved? How was this done?
14. What do the contacted populations depend on, or need to ensure inclusion in the services you offer?
15. What barriers or challenges prevent them from integrating?
16. How has the implementation of HIV testing services you offer been realized? How frequently is testing offered? Are people open to testing services? Why or why not?
17. How have people responded in cases of HIV positive test results? What do you tell the, or how do you respond, counsel?
18. Do you provide linkage to care or aid in a search for healthcare? How do you do this, how is it achieved?
19. What have you heard about the treatment that people receive when they get healthcare?
20. Is there coordination between the HIV clinics, centers and MINSAL? How does the coordination work?
21. When there are problems in coordination how are they resolved?
22. Are there any established relationships between your organization and the CCM? What kind or what nature?
23. What do you consider to be your achievements? Do they coincide with the objectives of the combined prevention strategy? How are they different?
24. Do you think the goals of the combined prevention strategy are realistic? Can they be reached/achieved? Reasons why or why not?
25. What have been the best experiences that you have had in this work? Which ones have been less positive or negative?
26. FOR SUPERVISORS: What’s your impression of the performance of the health promoters? Has there been employee turnover? Why, for what reasons? What recommendations would you offer to overcome any difficulties in the work of the health promoters?
